# Renal systolic time intervals derived from intra-renal artery Doppler as a novel predictor of adverse cardiac outcomes

**DOI:** 10.1038/srep43825

**Published:** 2017-03-07

**Authors:** Wen-Hsien Lee, Po-Chao Hsu, Chun-Yuan Chu, Szu-Chia Chen, Hung-Hao Lee, Meng-Kuang Lee, Chee-Siong Lee, Hsueh-Wei Yen, Tsung-Hsien Lin, Wen-Chol Voon, Wen-Ter Lai, Sheng-Hsiung Sheu, Po-Lin Kuo, Ho-Ming Su

**Affiliations:** 1 Graduate Institute of Clinical Medicine, College of Medicine, Kaohsiung Medical University, Kaohsiung, Taiwan; 2 Division of Cardiology, Department of Internal Medicine, Kaohsiung Medical University Hospital, Kaohsiung Medical University, Kaohsiung, Taiwan; 3 Department of Internal Medicine, Kaohsiung Municipal Hsiao-Kang Hospital, Kaohsiung Medical University, Kaohsiung, Taiwan; 4 Faculty of Medicine, College of Medicine, Kaohsiung Medical University, Kaohsiung, Taiwan

## Abstract

The aim of this study was to evaluate the use of renal systolic time intervals measured by electrocardiographic gated Doppler ultrasonography for predicting adverse cardiac events. This longitudinal observation study enrolled 205 patients. Renal systolic time intervals, including pre-ejection period (PEP) and ejection time (ET), and ratio of renal PEP to ET, were measured by electrocardiographic gated Doppler ultrasound. The 14 adverse cardiac events identified in this population included 9 cardiac deaths and 5 hospitalizations for heart failure during an average follow up of 30.9 months (25^th^–75^th^ percentile: 30–33 months). Renal PEP (hazard ratio = 1.023, P = 0.001), renal ET (hazard ratio = 0.975, P = 0.001) and renal PEP/ET (per 0.01 unit increase, hazard ratio = 1.060, P < 0.001) were associated with poor cardiac outcomes. The addition of renal PEP/ET to a Cox model containing important clinical variables and renal resistive index further improved the value in predicting adverse cardiac events (Chi-square increase, 9.996; P = 0.002). This study showed that parameters of intra-renal hemodynamics were potential predictors of adverse cardiac outcomes. However, the generalizability of these indicators need to be investigated in future large-scale studies.

Heart failure is an important clinical issue associated with high morbidity, high cardiovascular (CV) mortality and high health care costs^1^. In hospitalized patients with acute decompensated heart failure, deterioration of renal function is associated with adverse cardiac prognosis^2^. In clinical practice, cardiac and renal dysfunction have synergistic effects that aggravate poor cardiac and renal outcomes^3^. Although estimated glomerular filtration rate is widely used as a serum marker for evaluating renal function, parameters of renal Doppler ultrasound used to analyze intra-renal hemodynamics are independent indicators of CV outcome^4^. Renal resistive index (RI), which is an established indicator obtained from Doppler ultrasound spectra for intra-renal arteries, reflects renal vascular resistance and can predict decline in renal function, pathological change in renal parenchyma, and adverse CV events^4^,^5^,^6^,^7^. In fact, renal RI correlates with left ventricular diastolic dysfunction but not with ventricular ejection fraction (LVEF)^8^,^9^.

The impairment of left ventricular systolic function is an important predictor of heart failure^10^,^11^. In addition to LVEF, other widely used global cardiac performance and prognostic predictors in patients with heart failure include cardiac systolic time interval (STI), including pre-ejection period (PEP), ejection time (ET), and ratio of PEP to ET^12^,^13^,^14^. Prolonged cardiac PEP, prolonged PEP/ET, and short ET are associated with decreased LV systolic function^15^,^16^,^17^. Our recent study showed that renal STIs measured by Doppler ultrasonography were significantly associated with cardiac STIs, which suggests that renal STIs may be associated with adverse CV outcomes^18^. However, no studies have used a single imaging modality to investigate the relationship of intra-renal hemodynamics and adverse cardiac events. Therefore, we hypothesized that renal STI measured by real-time internal electrocardiographic gated Doppler ultrasonography can predict adverse CV outcomes.

## Methods

### Study subjects and design

**Figure 1:**
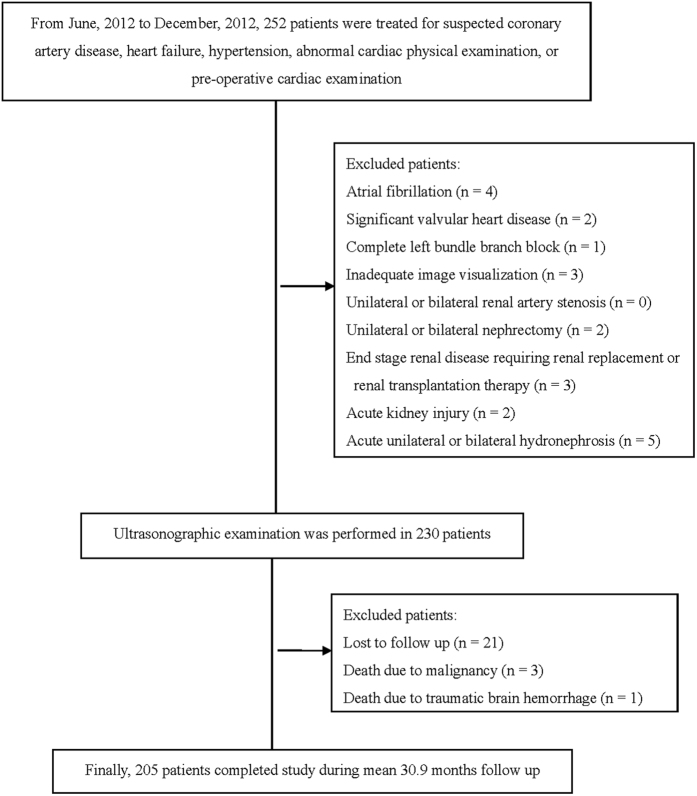
Flowchart of recruitment procedure.

This longitudinal observational study enrolled 252 participants who had received ultrasonographic examination in a regional hospital in Taiwan from June, 2012 to December, 2012. Excluded patients (n = 22) included those with atrial fibrillation, significant valvular heart disease, left bundle branch block, or inadequate image visualization. Patients were also excluded if they had any history of the following: unilateral or bilateral renal artery stenosis, unilateral or bilateral nephrectomy, end stage renal disease requiring renal replacement or renal transplantation therapy, acute kidney injury, or acute unilateral or bilateral hydronephrosis. Twenty-one patients were lost to follow up. Three died of malignancy, and one had traumatic brain hemorrhage. Figure 1 shows that 205 patients completed the study.

### Ethics statement

The study methods were carried out in accordance with the approved guidelines. The study protocols were approved by the institutional review board committee of the Kaohsiung Medical University Hospital (KMUHIRB-E(I)-20150180). Written informed consent was obtained from all subjects.

### Renal Doppler ultrasonography study

Renal ultrasonographic examinations were performed with a CX50 machine (Philips Compact Xtreme System, USA). Renal RI, renal PEP, and renal ET were measured as described in our previous study^18^. Briefly, renal RI was measured as (peak systolic velocity – minimum diastolic velocity)/peak systolic velocity from arcuate arteries in intra-renal Doppler. Renal PEP was determined from the beginning of the electrocardiographic QRS complex to the foot of the intra-renal pulse Doppler signal. Renal ET was determined from the foot to the dicrotic notch of the intra-renal pulse Doppler signal. Three measurements were taken for the kidney on each side, and the mean value for each kidney was recorded for further analysis.

### Collection of demographic, medical, and laboratory data

Baseline medical history and laboratory test values were collected from medical records. The eGFR was calculated by the equation used in the Modification of Diet in Renal Disease study^19^.

### Definition of cardiac events

The cardiac events were defined as cardiac death and hospitalization for acute decompensated heart failure. Surviving patients were followed up until June, 2015.

### Statistical analysis

Baseline data were presented as percentage or mean ± standard error. The predictors of cardiac events (cardiac death and hospitalization for heart failure) were analyses by Cox proportional hazards model. Time to cardiac events and covariates of risk factors were modeled using Cox proportional forward hazards model. In terms of their use for evaluating risk of adverse cardiac events, incremental values for renal PEP/ET were compared with conventional parameters by calculating the improvement in global Chi-square. Kaplan-Meier survival analysis with log-rank test was performed to determine the predictive role of renal PEP/ET in adverse cardiac outcomes. A P value less than 0.05 was considered statistically significant. Statistical analysis was performed using SPSS version 18.0 (SPSS Inc., Chicago, IL, USA).

## Results

A total of 205 participants enrolled in this study. Table 1 shows the clinical and renal Doppler ultrasonographic characteristics of these patients. The mean age was 64.4 ± 12.3 years, and 59.5% of participants were male. In renal Doppler parameters, the mean values for renal RI, renal PEP, renal ET, and renal PEP/ET were 0.69 ± 0.084 ms, 123.3 ± 23.7 ms, 304.8 ± 36.7 ms, and 0.41 ± 0.11, respectively. The variability coefficients of renal RI, renal PEP, renal ET, and renal PEP/ET were 0.12, 0.19, 0.12, and 0.27, respectively.

The follow-up period was 30.9 months (25^th^–75^th^ percentile: 30–33 months). During the follow-up period, 14 cardiac events occurred, including 9 deaths and 5 hospitalizations for heart failure. Table 2 shows the results of a Cox proportional hazards regression analysis of cardiac events. Univariable analysis showed that increased cardiac events were significantly associated with the presence of diabetes mellitus and chronic heart failure, increased heart rate, increased serum glucose, decreased estimated glomerular filtration rate, decreased hemoglobin, use of diuretics and β blockers, increased renal RI, increased renal PEP (hazard ratio [HR] 1.023; 95% confidence interval [CI] 1.010 to 1.037; P = 0.001), decreased renal ET (hazard ratio = 0.975; 95% CI = 0.961 to 0.989; P = 0.001), and increased renal PEP/ET (per 0.01 unit increase, HR = 1.060; 95% CI = 1.034 to 1.088; P < 0.001).

**Figure 2:**
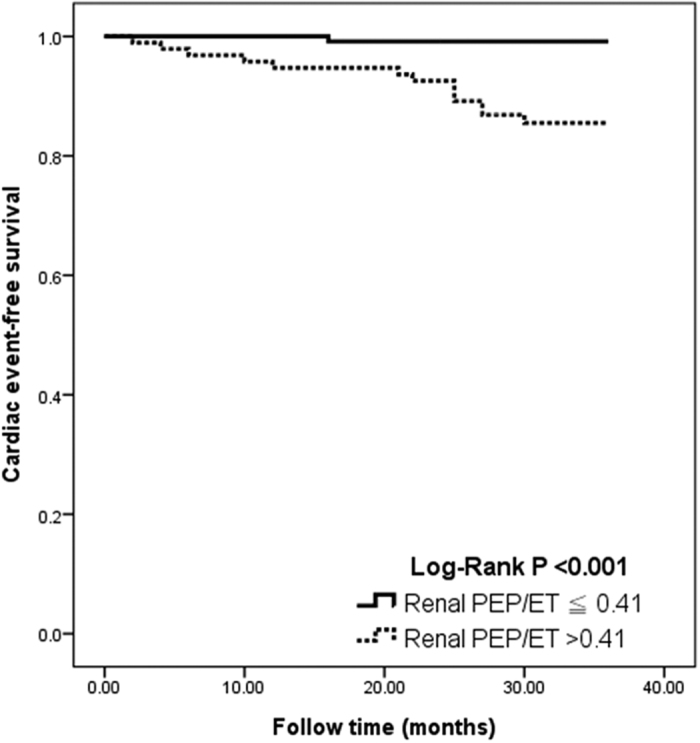
Results of Kaplan-Meier analysis of cardiac event-free survival in study patients.

To find the appropriate cut-off value for using renal PEP/ET to predict adverse cardiac events, several models were constructed using different cut-off values. A comparison of Chi-square values showed that the renal PEP/ET >0.41 model was the best predictor of adverse cardiac events. Figure 2 compares Kaplan-Meier curves for cardiac event-free survival between renal PEP/ET 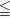 0.41 and renal PEP/ET >0.41 (log-rank P < 0.001).

**Figure 3:**
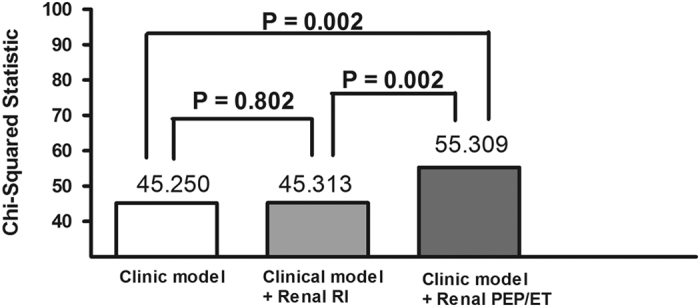
Addition of the ratio of renal pre-ejection period (PEP) to ejection time (ET) significantly improved prediction of adverse cardiac events in the basic clinical model (diabetes mellitus, chronic heart failure, serum glucose, estimated glomerular filtration rate, hemoglobin, and diuretic and β blocker use) and in the basic clinical model with the addition of renal resistive index (RI) (both P = 0.002).

Figure 3 shows how incremental change in renal PEP/ET is related to CV outcome. The clinical model consisted of variables potentially related to adverse CV outcomes in univariable analysis. These variables included presence of diabetes mellitus, chronic heart failure, serum glucose, estimated glomerular filtration rate, hemoglobin, and diuretic and β blocker use. In the clinical model, these variables were significant predictors of adverse cardiac events (Chi-square = 45.250, P < 0.001). However, prediction of adverse cardiac events did not significantly differ between the clinical model and the clinical model plus renal RI (Chi-square = 45.313, P = 0.802). Compared with the clinical model and the clinical model plus renal RI, the clinical model plus renal PEP/ET (Chi-square = 55.309) had significantly higher value in predicting adverse cardiac events (both P = 0.002).

## Discussion

This study showed that parameters of renal systolic time intervals derived by Doppler ultrasound are associated with adverse cardiac outcome. Notably, increased renal PEP/ET had significantly higher incremental prognostic value compared to conventional clinical parameters for predicting adverse cardiac events.

The LVEF is widely used to assess left ventricular systolic function^17^. An alternative parameter for evaluating cardiac systolic performance is STI^20^, Patients with decreased LVEF and left ventricular contractility have a long PEP, a shortened ET, and a long PEP/ET^15^,^20^,^21^. Various STIs measured by non-invasive sphygmography, phonocardiography, peripheral arterial waveform recordings, or echocardiography reportedly have significant correlations with LVEF^16^,^17^,^22^,^23^,^24^,^25^. Clinical applications of STI have been reported in various cardiac diseases, including heart failure, coronary artery disease, and hypertension^14^,^26^,^27^. The STI not only predicts global cardiac systolic function, it also predicts adverse CV events. Our previous studies showed that brachial PEP/brachial ET has a significant correlation with adverse CV outcomes in patients with hemodialysis and chronic kidney disease^28^,^29^. In the present study, renal STI derived by electrocardiographic gated renal Doppler ultrasound were associated with adverse CV outcomes. Compared to conventional clinical parameters, renal PEP/ET could be a superior predictor of cardiac prognosis.

Atherosclerotic reno-vascular disease is associated with an increased risk of death, cardiovascular events, and hospitalization^30^. Renal RI measured by Doppler ultrasound is a useful parameter for evaluating renal function and intra-renal vascular hemodynamics. Renal RI can be used not only to assess renal vascular resistance, but also to predict renal and CV outcomes^31^,^32^. Ennezat *et al*. demonstrated the use of renal RI as an independent predictor of re-hospitalization for heart failure in heart failure patients with preserved LVEF^31^. Ciccone *et al*. further showed that renal RI is an independent incremental predictor of disease progression in heart failure patients with reduced LVEF^33^. In the present study, renal RI showed significant association with CV outcome in the univariable analysis. However, renal RI did not have higher incremental prognostic value compared to conventional clinical parameters for predicting poor CV outcomes. The major difference between our study and previous studies of the relationship between renal RI and heart failure hospitalization was the study population. For example, Ennezat *et al*. and Ciccone *et al*. analyzed heart failure patients who had preserved or reduced LVEF. In contrast, we included patients referred for echocardiographic examinations, and only a small percentage (17.6%) of patients had a history of heart failure. Differences in the study population may partially explain the inconsistent results.

## Study limitations

This study had several limitations. First, this observational study had a longitudinal design and was limited to a relatively small number of cases in a regional hospital, which might limit the generalizability of the results. Furthermore, the number (n = 14) of adverse cardiac events was too low to conduct meaningful multivariable analyses in the study. Second, most of our patients had been treated with medications for arterial hypertension. For ethical reasons, these medications could not be withdrawn. Hence, their effects on the present findings could not be completely excluded. Third, since the subjects of this study were already being evaluated for possible cardiac disease by echocardiography, the study was susceptible to selection bias, which also reduces the generalizability of the findings. Finally, patients with a history of renal artery stenosis, nephrectomy, end stage renal disease requiring renal replacement or renal transplantation therapy, acute kidney injury, and hydronephrosis were also excluded. Therefore, our results are inapplicable in these patients.

## Conclusions

This study is the first to show that renal systolic time intervals were associated with adverse cardiac events and may be better than conventional clinical parameters for predicting cardiac prognosis. Hence, these parameters should be included in renal Doppler ultrasound examination to improve prognostication. However, the generalizability of these indicators need to be investigated in future large-scale studies.

## Additional Information

**How to cite this article**: Lee, W.-H. *et al*. Renal systolic time intervals derived from intra-renal artery Doppler as a novel predictor of adverse cardiac outcomes. *Sci. Rep.* **7**, 43825; doi: 10.1038/srep43825 (2017).

**Publisher's note:** Springer Nature remains neutral with regard to jurisdictional claims in published maps and institutional affiliations.

## Figures and Tables

**Table 1:** Clinical and renal Doppler ultrasonographic characteristics of study patients.

Characteristics	All patients (number = 205)
Age (years)	64.4 ± 12.3
Male gender (%)	59.5
Smoking (%)	20.0
Diabetes mellitus (%)	30.2
Hypertension (%)	73.2
CAD (%)	16.6
Stroke (%)	10.2
Heart failure (%)	17.6
Systolic BP (mmHg)	133.3 ± 17.8
Diastolic BP (mmHg)	75.4 ± 11.1
Pulse pressure (mmHg)	57.9 ± 12.6
Heart rate (min^−1^)	68.6 ± 11.7
Body mass index (kg/m^2^)	26.3 ± 3.8
Total cholesterol (mg/dL)	191.3 ± 41.0
eGFR (mL/min/1.73 m^2^)	60.9 ± 20.8
Glucose (mg/dl)	120.5 ± 43.5
Hemoglobin (g/dL)	13.4 ± 1.9
**Medications**	
ACEI (%)	18.0
ARB (%)	44.4
β-blocker (%)	44.9
CCB (%)	47.3
Diuretics (%)	35.1
**Renal Doppler ultrasound**	
Renal RI	0.69 ± 0.084
Renal PEP (ms)	123.3 ± 23.7
Renal ET (ms)	304.8 ± 36.7
Renal PEP/ET	0.41 ± 0.11

Abbreviations.ACEI: angiotensin converting enzyme inhibitor; ARB: angiotensin II receptor blocker; BP: blood pressure; CAD: coronary artery disease; CCB: calcium channel blocker; eGFR: estimated glomerular filtration rate; ET: ejection time; ms, millisecond; pre-ejection period; RI: resistive index.

**Table 2:** Predictors of cardiac events (cardiac death and hospitalization for heart failure) using Cox proportional hazards model.

Parameter	HR (95% CI)	*P* value
Age (year)	1.001 (0.959, 1.045)	0.949
Male gender (%)	1.264 (0.423, 3.771)	0.675
Smoking (%)	1.079 (0.301, 3.868)	0.907
Diabetes mellitus (%)	6.367 (1.996, 20.318)	0.002
Hypertension (%)	2.231 (0.499, 9.971)	0.293
CAD (%)	1.479 (0.412, 5.303)	0.548
Stroke (%)	0.693 (0.091, 5.297)	0.724
Heart failure (%)	38.017 (8.424, 171.565)	<0.001
Systolic BP (mmHg)	0.999 (0.969, 1.029)	0.928
Diastolic BP (mmHg)	1.025 (0.985, 1.067)	0.221
Pulse pressure (mmHg)	0.972 (0.927, 1.018)	0.228
Heart rate (min^−1^)	1.050 (1.010, 1.092)	0.013
Body mass index (kg/m^2^)	0.952 (0.820, 1.107)	0.525
Total cholesterol (mg/dL)	0.989 (0.972, 1.006)	0.203
eGFR (mL/min/1.73 m^2^)	0.941 (0.916, 0.966)	<0.001
Glucose (mg/dl)	1.010 (1.002, 1.018)	0.011
Hemoglobin	0.612 (0.457, 0.819)	0.001
**Medications**		
ACEI use (%)	0.336 (0.044, 2.566)	0.293
ARB use (%)	2.384 (0.799, 7.113)	0.119
β-blocker use (%)	4.822 (1.345, 17.293)	0.016
CCB use (%)	0.423 (0.133, 1.348)	0.146
Diuretics use (%)	4.965 (1.557, 15.837)	0.007
**Renal Doppler ultrasound**		
Renal RI (per 0.01)	1.093 (1.017, 1.173)	0.015
Renal PEP (ms)	1.023 (1.010, 1.037)	0.001
Renal ET (ms)	0.975 (0.961, 0.989)	0.001
Renal PEP/ET (per 0.01)	1.060 (1.034, 1.088)	<0.001

HR: hazard ratio; CI: confidence interval; other abbreviations as in Table 1.
